# Independent Emergence of the Cosmopolitan Asian Chikungunya Virus, Philippines 2012

**DOI:** 10.1038/srep12279

**Published:** 2015-07-23

**Authors:** Kim-Kee Tan, Ava Kristy D. Sy, Amado O. Tandoc, Jing-Jing Khoo, Syuhaida Sulaiman, Li-Yen Chang, Sazaly AbuBakar

**Affiliations:** 1 Tropical Infectious Diseases Research and Education Centre (TIDREC), University of Malaya, 50603 Kuala Lumpur, Malaysia; 2 Department of Medical Microbiology, Faculty of Medicine, University of Malaya, 50603 Kuala Lumpur, Malaysia; 3 Virology Department, Research Institute for Tropical Medicine, Department of Health, FCC Compound, Alabang, Muntinlupa City, Philippines

## Abstract

Outbreaks involving the Asian genotype *Chikungunya virus* (CHIKV) caused over one million infections in the Americas recently. The outbreak was preceded by a major nationwide outbreak in the Philippines. We examined the phylogenetic and phylogeographic relationships of representative CHIKV isolates obtained from the 2012 Philippines outbreak with other CHIKV isolates collected globally. Asian CHIKV isolated from the Philippines, China, Micronesia and Caribbean regions were found closely related, herein denoted as Cosmopolitan Asian CHIKV (CACV). Three adaptive amino acid substitutions in nsP3 (D483N), E1 (P397L) and E3 (Q19R) were identified among CACV. Acquisition of the nsP3-483N mutation in Compostela Valley followed by E1-397L/E3-19R in Laguna preceded the nationwide spread in the Philippines. The China isolates possessed two of the amino acid substitutions, nsP3-D483N and E1-P397L whereas the Micronesian and Caribbean CHIKV inherited all the three amino acid substitutions. The unique amino acid substitutions observed among the isolates suggest multiple independent virus dissemination events. The possible biological importance of the specific genetic signatures associated with the rapid global of the virus is not known and warrant future in-depth study and epidemiological follow-up. Molecular evidence, however, supports the Philippines outbreak as the possible origin of the CACV.

Chikungunya virus (CHIKV: *Togaviridae: Alphavirus*) is a zoonotic arbovirus. It causes debilitating disease that can incapacitate afflicted sufferers. CHIKV is likely to have emerged from Africa where it was found to be maintained in an enzootic transmission cycle involving non-human primates and arboreal *Aedes* mosquitoes^1^. The occasional enzootic spillover caused small localized outbreaks in rural areas of West Africa^1^. Whereas, in urbanized areas of endemic region, the virus is normally associated with the *Aedes* spp. human transmission cycle involving *Ae. aegypti* and *Ae. Albopictus.* There are three genotypes of CHIKV identified to date, namely West African, Asian, and East-Central-South-Africa (ECSA) genotypes, reflecting the initial geographical distribution of the viruses.

CHIKV was first isolated from Tanzania during a dengue-like epidemic in 1953^2^. In the past, CHIKV outbreaks were mostly geographically-restricted and had only occasionally been reported in Africa and Asia, notably the Southeast Asia (SEA) region^3^,^4^,^5^,^6^,^7^,^8^,^9^,^10^,^11^. The first widespread CHIKV outbreak swept across the Indian Ocean in concomitant with the emergence of the Indian Ocean Lineage (IOL) of ECSA genotype in Réunion Island during the 2005 outbreak^12^,^13^. Successful adaptation of the IOL strain in *Ae. albopictus* led to the expansion of the virus geographic presence into *Ae. albopictus* infested regions in Asia, Indian Ocean and Europe (Italy and France)^6^,^13^,^14^,^15^. A major outbreak involving the Asian CHIKV genotype occurred in the Caribbean region towards the end of 2013^16^,^17^,^18^. Subsequently, autochthonous transmissions of Chikungunya were documented in the Americas. As of 2014, there were more than 1,071,696 suspected cases recorded with 22,796 of these were laboratory confirmed^19^. Recent findings revealed close phylogenetic relationships between the emerging Caribbean Asian CHIKV with isolates collected from Indonesia, the Philippines, China and Micronesia prior to the occurrence of the Caribbean outbreak^18^. International travel between the Caribbean and CHIKV endemic regions was the likely contributing factor of the infection^20^. In particular, a major nationwide Asian genotype CHIKV outbreak occurred in the Philippines in 2012 highlighting the possibility that the Philippines was the origin of the Cosmopolitan Asian CHIKV^21^.

Here, we report the full-length genome sequencing and phylogeography of the CHIKV isolates collected from different provinces in the Philippines during the 2012 nationwide outbreak. By using the genome scale analysis, we explored the microevolution, genetic trait and phylogenetic relationships of the Philippines isolates with other old and the recently emerged Caribbean Asian CHIKV isolates. Result from the study illustrates the possible origin of current globally spreading Asian genotype CHIKV and improves the understanding of the epidemiological characteristic of the ongoing CHIKV outbreak in the Americas.

## Results

### Genetic diversity and geographical distribution of the Philippines CHIKV

**Figure 1:**
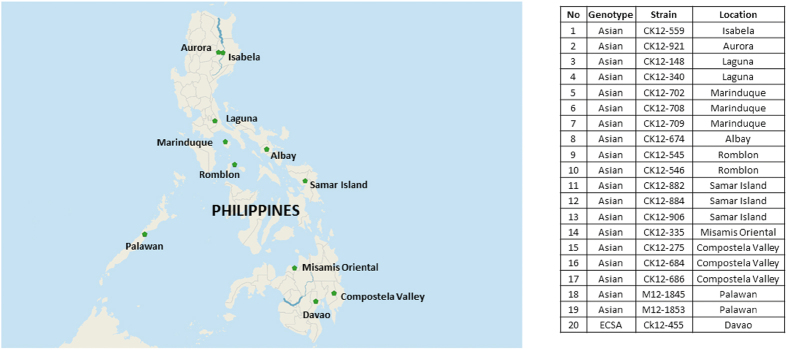
Geographical distribution of CHIKV isolated from the Philippines, 2012. The map was created with Natural Earth, free vector and raster map data @ naturalearthdata.co using QGIS Desktop 2.8.1^47^. This map is licensed under Creative Commons Attribution-ShareAlike 3.0 licence (CC BY-SA) http://creativecommons.org/licenses/by-sa/3.0/.

Genetic diversity of CHIKV circulating during the Philippines 2012 outbreak was determined using the partial E1 gene sequences. Nineteen CHIKV isolates representing distinct geographical locations were sequenced (Fig. 1). Using the genome sequences all the Philippines isolates were identified as belonging to the Asian genotype except CK12-455 (isolated from Davao), which grouped into the IOL within the ECSA genotype.

### Genome structure and molecular signatures of the Philippines Asian CHIKV

We determined nearly complete genome sequence (11542 to 11599 nucleotides) of 16 Philippines Asian CHIKV isolates, consisting of nucleic acids from position 38 at the 5′ non-translated region to position 11649 at the 3′ non-translated region of the CHIKV TH-35. All the Philippines isolates possessed two open reading frames in the coding region that encoded for structural (capsid, E3, E2, 6k and E1), non-structural (nsP1, nsP2, nsP3 and nsP4) polyproteins and an opal stop codon at nsP3 codon 524.

Pairwise comparison of the Philippines Asian genotype CHIKV sequences revealed a unique deletion of 48 nucleotides (11361 to 11408) at the 3′ non-translating region in all isolates collected from Mariduque (CK12-702, CK12-708 and CK12-709). Within the protein-coding region, 29 amino acid substitutions were observed (Table 1). Twelve of the amino acid substitutions were located within the structural polyproteins and 17 within the non-structural polyproteins, resulting in 0.96% and 0.69% amino acid changes, respectively.

Among the amino acid substitutions, nsP4-107H, nsP4-110S and E2-11V were specific for all isolates collected from Marinduque; nsP3-351D, nsP4-366 S and 6K-46M were unique to all isolates collected from Samar Island. The nsP3-88V and nsP4-101A were detected in isolates collected from Compostela Valley (CK12-684 and CK12-686); the latter was also detected in isolate from Aurora (CK12-921). The E2-297H was detected in samples collected from Romblon and Misamis Oriental. The E3-19R and E1-397L were specific to all the Philippines isolates except those collected from Aurora, Compostela Valley and Laguna (CK12-340).

### Selection pressure analysis of complete coding region of Asian CHIKV

The selection pressure on all protein-coding genes of Asian CHIKV was examined using HyPhy package as implemented in Datamonkey server. Based on the GARD analysis, there was no recombination in all the sequence alignments. Based on the PARRIS method, no evidence of positive selection was observed in sequence alignments of Asian genotype CHIKV. We observed positive selection codon by using the codon specific selection methods, where IFEL, REL and MEME identified 45 positive selection sites (Tables 2 and 3). Nine and thirty-six positively selected sites were identified in structural and non-structural genes, respectively. Three residues were identified as positively selected site by two out of three abovementioned methods. The nsP3-77 was identified by REL (Bayes factor = 434.6) and MEME (p = 0.034); nsP3-451 was identified by IFEL (p = 0.083) and REL (Bayes factor = 1069.35); and nsP4-81 was identified by REL (Bayes factor = 1770.92) and MEME (p = 0.007).

### Molecular signature and phylogenetic relationship of Asian CHIKV

**Figure 2:**
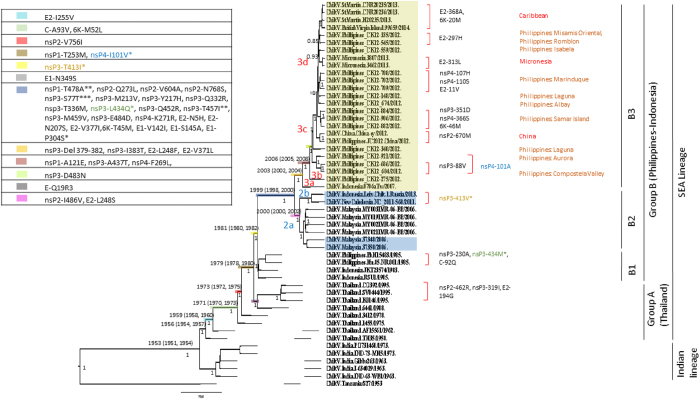
Maximum clade credibility (MCC) tree of CHIKV. The phylogenetic tree was constructed using partial sequence of E1 genes. The three genotypes of CHIKV, ECSA, Asia and West Africa were shown. The estimated 95% HPD values for the most recent common ancestors and the Bayesian posterior probability values were indicated adjacent to the node. For clarity, the CHIKV Asian clade containing strains isolated after 2006 is shown enlarged in the inset at the upper left.

The relationship of the Asian CHIKV, collected globally, was examined using the phylogenetic tree reconstructed with open reading frame regions of the CHIKV genome (11,237 nucleotides). From the phylogenetic tree, the isolates of Asian genotype segregated into two major clades, the Indian and the SEA lineages (Fig. 2). The Indian lineage merely consisted of CHIKV isolates from India (Fig. 2), and the SEA lineage comprised isolates obtained from CHIKV endemic countries in SEA (Thailand, Indonesia, Malaysia, the Philippines), the newly emerged Cosmopolitan Asian CHIKV (CACV) strains from China, Oceania (New Caledonia and Micronesia) and Caribbean (Saint Martin and British Virgin Island). The TH35 strain isolated from Thailand in 1958 occupied the ancestral node of the SEA lineage in the phylogenetic tree, suggesting that segregation of Indian and SEA lineages occurred earlier than 1958. This is in agreement with our analysis where the Indian and SEA lineages shared the most recent common ancestor (MRCA) that could have circulated in this region dating back to 1953 (95% HDP:1951 to 1954).

Subsequently, deduced amino acid sequences of the SEA Asian CHIKV were examined. Simultaneous with the phylogenetic and geographical analyses, we determined that sequential amino acid substitutions occurred during the evolution of SEA CHIKV lineages. These changes reflected the evolutionary path of the CHIKV in SEA region and the recent emergence in the Caribbean area. The representative informative sites of the sequential amino acid changes and geographic clade specific sites are shown in Fig. 2. Within the SEA lineage, the isolates segregated into clades in similar spatial and temporal patterns as previously reported^22^. Distinct clustering between the Thai and Philippines-Indonesia isolates in the phylogenetic tree was observed. The Thai isolates was denoted group A (SEA mainland isolates); the Philippines-Indonesia isolates and its descendant were denoted group B (SEA Island isolates). The MRCA analysis suggested that this divergence could have been dated back to 1981 (95% HDP: 1980 to 1982). Amino acid substitution from threonine to isoleucine at nsP3 position 413 was first observed in the Philippines-Indonesian clade among isolates from 1983. The substitution was inherited by descendant in group B except NC_2011-568 and Leiv.Chik.1, which possessed valine at the position. The substitution was absent in all of the Thai isolates collected so far (until year 1995), supporting our MRCA estimation that the virus diverged into the SEA mainland (Thai) and SEA island (Philippines-Indonesian) clades started as early as 1980s.

The SEA island isolates in clade B continued to evolve and can be further delineated into three major clades, B1, B3 and B3 which corresponded to their distinct spatial and temporal characteristics. The B1 clade represented the old Philippines-Indonesia CHIKV obtained during 1980s, which become extinct. B2 and B3 were the most recent circulating Asian CHIKV strains evolved from the B1 clade. According to the MRCA estimation, B2 and B3 clades shared a common ancestor that had been circulating in the regions since 15 years ago (95% HDP: 1998 to 2000). We identified 22 sequential amino acid substitutions spanning the CHIKV genome that represented the B2/B3 common ancestral strains prior to the predicted divergence in 1999 (Fig. 2). Among the 22 evolutionary associated substitutions, two of them were not inherited in all B2/B3 isolates. The nsP3-77 from serine to threonine was absent in NC-2011-568, Leiv.Chik.1 and 0706Tw, while the nsP3-457 substitution from threonine to isoleucine was absent in isolate 0706Tw. All three isolates were reported to have Indonesian origin.

The B2 clade demonstrated a restricted geographical distribution and only consisted of isolates collected from local outbreaks in Malaysia (2006), New Caledonia (2011), and a single isolate from Russia (traveler back from Indonesia). The B2 clade possessed serine at position 248 of E2 protein and valine at position 486 of nsP2 protein, which are clade-specific amino acid substitutions that differentiated them from other SEA Asian CHIKV isolates. Within the B2 clade, the isolates formed geographical clades where all the Malaysian isolates grouped and formed a sub-clade herein named B2a. While the isolates in common with the Indonesian origin (NC_2011-568 and Leiv.CHIKV.1) clustered within the sub-clade B2b. Both of the isolates possessed valine at nsP3-413, which was a unique signature to B2b sub-clade. Result from MRCA analysis suggested, these two sub-clades diverged in year 2000 (95% HDP: 2000 to 2002). Deletion of seven amino acids (376-382) in nsP3 protein was detected in four out of eight isolates in B2 clade.

The B3 clade was the only cosmopolitan Asian CHIKV clade with isolates found geographically dispersed. We identified four unique molecular signatures of the B3 clade: nsP3-Del 379-382, nsP3-I383T, E2-L248F, E2-V371L that confirmed the emergence of B3 clade from a common ancestor dated to 2003 (95% HDP: 2002 to 2004). All the Philippines CHIKV isolated from the nationwide CHIKV outbreaks in 2012 grouped into this clade. Other isolates are CACV described from China (2012), Micronesia (2013) and the Caribbean region (2013–2014) descended from the common ancestor of the B3 clade viruses. By using the unique amino acid substitutions observed, we further classified the virus into several sub-clades (B3a-d). Indonesian isolates, CHIKV 0706aTW (B3a) rooted at the basal of B3 clade suggested the origin of B3 from Indonesia. However, the clade remained silent after the isolation of 0706aTW until the 2012 Philippines outbreak. Analysis of the genome sequences of 25 B3 clade CHIKV revealed additional three amino acid substitutions: nsP1-A121E, nsP3-A437T, nsP4-F269L that were present in all isolates in B3b-d sub-clades compared to the 0706aTW (B3a). We identified two additional evolutionary-associated amino acid substitutions that allowed differentiation of sub-clades B3b, B3c, and B3d. The substitution nsP3-D483N were present in all B3c/B3d isolates. While the substitution of E3-Q19R was unique to all isolates in sub-clade B3d.

CACV described from China, Micronesia and the Caribbean region clustered spatially with viruses identified from the same geographical location and formed a distinct geographic clade. The geographic clades were interspersed within the Philippines monophyletic clade (Fig. 2). The two China isolates (JC2012 and China-sy) with SEA origin (likely the Philippines) clustered together and fell into sub-clade B3c. While the Micronesian and Caribbean isolates clustered into sub-clade B3d. The Micronesian isolates grouped closely with CK12-559 identified from Isabela Island in the Philippines. Whereas, the Caribbean CHIKV (Saint Martin and British Virgin Island) clustered with CK12-545 and CK12-335 identified from Romblon and Misamis Oriental group of islands, respectively. This suggested independent emergence of CHIKV from the Philippines, which then spread into China, Micronesia and the Caribbean region.

## Discussion

Rapid CHIKV dissemination driven by *Aedes albopictus*-adapted IOL lineage was reported in many *Aedes albopictus* infested countries^22^,^23^,^24^,^25^. Epidemiological evidence suggests that through competitive displacement^26^, the emergence of IOL strain in SEA region may eventually lead to the extinction of the endemic Asian genotype, which has mainly circulated locally in the region over the past six decades. While it was true that most outbreaks involving the Asian genotype CHIKV were limited and localized, the continuous reporting of the virus in Indonesia during 2008 to 2009 and New Caledonia in 2011 points to a resilient presence of the Asian genotype CHIKV in Island SEA and Pacific Oceania regions^3^,^27^,^28^,^29^. Resurgence of Asian genotype virus in the Philippines beginning in 2011 accentuated the rise of a new CHIKV epidemic in the country after 20 years of quiet inter-epidemic period^21^. This nationwide outbreak provides additional evidence to support the continuous endemicity of the Asian genotype CHIKV in SEA region. It is noted that unlike other SEA countries, Philippines was not affected by the contagion involving the IOL ECSA virus when the epidemic swept the region in 2012^6^,^10^,^11^,^14^,^30^. Only a single ECSA isolate was obtained for our study and this was from Davao a major port city in the Southern Philippines nearing East Malaysia which had reported large surge of CHIKV infection during 2010^31^,^32^. The ongoing CHIKV outbreaks in the Caribbean and Micronesia were caused by Asian CHIKV strains^18^. Our phylogenetic data was in agreement with recent report and indicated the outbreak causing strains were closely related to the Philippines isolates responsible for the 2012 CHIKV outbreaks^18^. In contrast to the geographically restricted feature of previous Asian genotype-associated CHIKV outbreaks, the current Asian genotype CHIKV epidemic/outbreaks demonstrated wide dispersing characteristic that resemble the IOL-associated outbreaks in Indian Ocean area^13^.

By incorporating the phylogeographical analysis and microevolution on the virus genome, we suggest a possible scenario contributing to the global spread of the CACV. Deletion of similar regions of the hypervariable C-terminal domain in the nsP3 gene was observed in most of the Asian genotype CHIKV isolated after 2006^33^,^34^. Considering the phylogenetic analysis and temporal distribution of the SEA isolates, our finding herein suggests that the new SEA CHIKV Asian strains have evolved from a common ancestor descended from an ancestral strain of the old Asian lineage (B1 clade) that possibly diverged during the period of 1998 to 2000 (95% HDP). Acquisition of the clade unique molecular signature in the C-terminal domain of the nsP3 gene may have occurred independently in Malaysia (nsP3_376 -382del_, B2 clade) and Indonesia (nsP3_379 –382del_, B3 clade). This could occur as a result of genetic convergence arisen from evolutionary adaptation to the local setting^33^. The C-terminal domain is an important determinative factor for optimal virus replication in various host cells in Alphavirus. The nsP3_376 –382del_ was identified in two out of six CHIKV isolates described from Malaysia in 2006, indicating the possibility of adaptive mutations during the localized outbreak. Prolonged circulation of Asian genotype CHIKV in Indonesia^28^,^29^ and ancestral location of the Indonesian isolate in B3 clade support the Indonesian origin of the B3 clade. This may ascribed an important evolutionary event that leads to transmission and spatial distribution of CHIKV in the SEA region. In addition, the amino acid substitution at position 248 in E2 protein is a clade-specific adaptive mutation that allowed the differentiation of the B2 (serine) and B3 (phenylalanine) clades. CHIKV E2 protein is important for virus attachment. The codon 248 is located at the acid-sensitive region of E2 protein that underwent positive selection (p-value ≤0.1). A recent study demonstrated that the mutation of E2-L248Q was beneficial for the dissemination of the virus in *Aedes albopictus*^35^. Whether the naturally occurred amino acid substitution of E2-248 would have beneficial effect on the viral fitness in *Aedes aegpti*, the mosquito vector in the Philippines and Caribbean region, warrants further investigation.

Traceable microevolution of the viral genome gives rise to the probable transmission route of virus^13^. Sequence analysis of the Philippines CHIKV revealed the acquisition of two unique adaptive amino acid substitutions, the nsP3-D483N, and E3-Q19R during the Philippines outbreaks. An amino acid substitution of Aspartic acid to Asparagine at nsP3 position 483 was present in all strains in B3 clade except the Philippines CK12-275 and Indonesian 0706aTW CHIKV. Whereas, the amino acid substitution of Glutamine to Arginine at E3 position 19 was concomitant with the nsP3-483N and was observed in all virus isolates from the Philippines except isolates collected from Compostela Valley and Aurora. Besides, an additional amino acid substitution of Proline to Leucine at E1 position 397 was observed in all E3-19R-bearing strains and the two China (JC2012 and China-sy) strains collected in 2012. However, the E1-397L was not a unique adaptive substitution observed in the Philippines outbreaks, as it is present in all Indian lineage Asian CHIKV. In the SEA lineage, the E1-397L was only observed in one of the Malaysian isolate, MY021IMR which was isolated from the 2006 outbreak in Bagan Panchor. Whether this substitution happened convergently in Malaysia and the Philippines remained inconclusive. This observation, however, points to unique sequential adaptive mutations of nsP3-483D/E1-397L/E3-19R that accumulated in the viral genome during the localized outbreaks.

Data from this study showed that the Philippines outbreaks could have begun from Compostela Valley, where the ancestral strain nsP3-483D co-existed with variants, nsP3-483N CHIKV. The variant, nsP3-483N strains spread into Laguna and Aurora prior to the acquisition of E3-19R and E1-397L. The unique amino acid substitution of nsP4-V101A was only observed in strains from Compostela Valley (CK12-684 and CK12-686) and Aurora (CK12-921), but not in Laguna strain (CK12-340) suggests that the spread into Laguna happened earlier than Aurora, which is prior to the acquisition of nsP4-101A. The co-existence of E3-19Q/E1-397P (CK12-340) and E3-19R/E1-397L (CK12-148) CHIKV in Laguna suggests that the acquisition of Arginine in E3 protein and Leucine in E1 protein could have happened by local adaptation. On the other hand, the detection of ‘intermediate’ strains (JC2012 and China-sy), bearing only nsP4D and E1-397L but not the E3-19Q, suggests the acquisition of E1-397L occurred prior to the E3-19R. These ‘intermediate’ strains could have circulated transiently in a restricted local setting (perhaps Laguna only) and then spread into China prior to the acquisition of the third amino acid substitution at E3-19Q. Thus far, the nsP3-483N/E1-397L/E3-19R-bearing strains have the most diverse geo-distribution. Rapid nationwide spread in the Philippines (Albay, Isabela, Marinduque, Misamis Oriental, Romblon and Samar Island) and globally (Micronesia and Caribbean) following acquisition of the third amino acid substitution (E3-19R).

Unlike the CHIKV epidemic involving the Asian genotype during the 1970 to 1990, the risk of widespread outbreak remains transitory as the human-*Aedes aegypti* interaction still lacks outbreak sustainability potential at a local scale, therefore requiring continuous virus importation^36^. Although the B3 clade appeared as early as 2007, high magnitude and rapid dissemination of the virus was only observed during the 2012 CHIKV outbreaks in the Philippines (acquisition of nsP3-483N) and later during its nationwide and global spread (acquisition of E1-397L and E3-19R). Out of the three unique adaptive amino acid substitution, two (nsP3-483 and E1-397) were predicted as positively selected sites. Currently, it is still unclear if these amino acid substitutions at nsP3-483, E1-397 and E3-19 are products of CHIKV adaptation to the *Aedes aegypti* in local transmission setting. Sequential acquisition of mutations that could have provided beneficial effect to the viral fitness has been previously demonstrated^35^. E3 protein is suggested to stabilize the interaction of E1/E2 protein by clamping the acid sensitive region of E2 in place until furin cleavage^37^. A recent study demonstrated the substitution of E3-S18F stabilized the E2 protein with E2-198^35^. Whether, the amino acid substitution of E3-19 exerts the same effect, remains to be investigated. It is possible that these mutations confer the newly emerging CHIKV with the ability to sustain epidemic human-*Aedes aegypti* transmission cycle.

The microevolution history of CHIKV genome reported herein suggests the Philippines as the possible origin of CACV causing the outbreaks in the Caribbean. Unique amino acid substitutions observed among the CACV suggests multiple independent virus dissemination events contributing to the global spread. While the possible biological importance of these mutations are still unknown, the genetic signatures identified in the study represent interesting candidates for future in-depth study and epidemiological follow-up. Sequencing of additional isolates from the outbreak regions and local reservoirs would allow better delineation of the evolution pattern of this globally emerging Asian CHIKV.

## Methods

### Virus strains

The WHO Collaborating Centre for Arbovirus Reference & Research (Dengue/Dengue Haemorrhagic Fever) at Tropical Infectious Diseases Research and Education Centre (TIDREC), University of Malaya received 27 serum samples from the Research Institute for Tropical Medicine, the Department of Health, Philippines. These serum samples were collected in 2012 from different provinces in the Philippines that represent the circulating viral strains during the 2012 outbreaks in the country.

### Sample preparation, genome sequencing and assembly

All laboratory activities involving the virus isolates was conducted following BSL-2 biosafety practices and procedures in BSL-2 laboratory. Viral RNA was extracted and screened for the CHIKV by using Reverse-transcription PCR (RT-PCR) previously described^6^. The E1 (N = 19) genes of positive sample were subsequently sequenced and analyzed to determine the virus genotype. Isolates identified as Asian genotype (N = 18) were subjected to full genome sequencing using the Ion Torrent sequencing platform (Life Technologies, USA). The raw sequence reads were assembled using the GSNAP algorithms as implemented in Sequencher V5.2.2^38^.

### Phylogenetic analysis

The genome sequences (Supplementary Table 1) were aligned using ClustalX 2.1^39^. The phylogeny, and the divergence time (tMRCA) of Asian CHIKV was estimated simultaneously using Bayesian Markov chain Monte Carlo approach in BEAST 1.8.0^40^ with Generalised time-reversible model with gamma distribution as selected by jModel Test 2.1.4^41^.

### Selection pressure analysis

The sequence alignment of individual gene (nsP1, nsP2, nsP3, nsP4, C, E3, E2, 6K and E1) for 49 CHIKV were analyzed using HyPhy package^42^ implemented in the Datamonkey server^43^ as previously described^44^. Prior to analysis, the sequence alignment was checked for duplication of genome sequences, which was removed upon identification. GARD^45^ method was used to screen for potential recombination in the dataset prior to the selection pressure analysis. The Parris method^46^ was used to assess the selection in the sequence alignment. While the specific site selection on each individual gene was analyzed using SLAC, FEL, IFEL, REL, FUBAR and MEME algorithm. Positive selection was defined as p-value ≤ 0.1 for PARRIS, SLAC, FEL, IFEL, MEME; Bayes factor ≥50 for REL or Posterior probability ≥0.9 for FUBAR.

## Additional Information

**Accession codes:** All genome sequences generated in this study are available from the European Nucleotide Archive with study accession number PRJEB8862 ( http://www.ebi.ac.uk/ena/data/view/PRJEB8862).

**How to cite this article**: Tan, K.-K. *et al.* Independent Emergence of the Cosmopolitan Asian Chikungunya Virus, Philippines 2012. *Sci. Rep.* **5**, 12279; doi: 10.1038/srep12279 (2015).

## Supplementary Material

Supplementary Information

## Figures and Tables

**Table 1:**
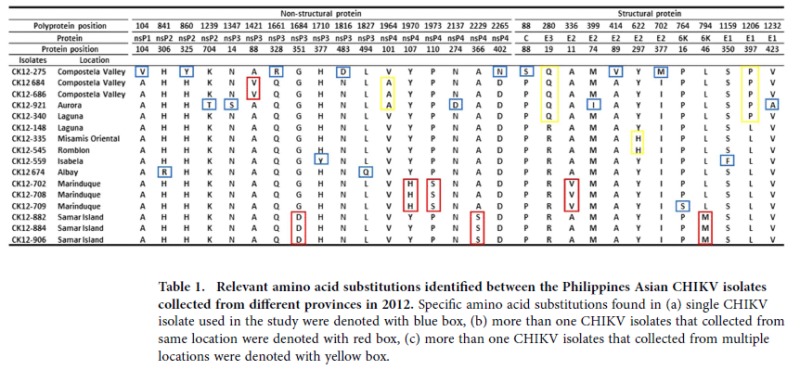
Relevant amino acid substitutions identified between the Philippines Asian CHIKV isolates collected from different provinces in 2012.

Specific amino acid substitutions found in (a) single CHIKV isolate used in the study were denoted with blue box, (b) more than one CHIKV isolates that collected from same location were denoted with red box, (c) more than one CHIKV isolates that collected from multiple locations were denoted with yellow box.

**Table 2:** Selection pressure analysis of Asian genotype Chikungunya complete ORF dataset (n = 49) using the SLAC, FEL, REL, FUBAR and MEME methods implemented in Datamonkey server (www.datamonkey.org).

**Dataset**	**Codon**	**SLAC**		**FEL**		**IFEL**		**REL**		**FUBAR**		**MEME**	
		**dN-dS**	**p-value**	**dN-dS**	**p-value**	**dN-dS**	**p-value**	**dN-dS**	**Bayes Factor**	**dN-dS**	**Post. Pr.**	**ω**^**+**^	**p-value**
NSP2 (n = 31)	67	16.35	0.667	77.74	0.233	152.792	0.137	−0.923	1	0.467	0.604	> 100	0.038
	768	1.012	0.308	78.2	0.173	151.984	0.097	−0.923	1	0.38	0.609	> 100	0.373
NSP3 (n = 37)	13	10.316	0.708	31.985	0.403	0	0	0.269	50.357	0.134	0.586	> 100	0.361
	14	10.155	0.701	30.678	0.291	0	0	0.295	52.473	0.151	0.598	> 100	0.417
	72	10.155	0.701	31.128	0.289	0	0	0.296	52.745	0.156	0.599	> 100	0.41
	**77**	**21.704**	**0.44**	**34.938**	**0.324**	**66.716**	**0.197**	**0.382**	**434.6**	**0.363**	**0.712**	**> 100**	**0.034**
	224	17.868	0.635	36.169	0.428	71.3	0.286	0.359	322.018	0.295	0.698	> 100	0.342
	238	10.155	0.701	26.678	0.247	48.763	0.156	0.306	51.228	0.143	0.607	> 100	0.401
	328	16.656	0.598	57.019	0.288	0	1	0.286	58.836	0.603	0.639	> 100	0.258
	332	13.13	0.629	36.162	0.35	69.991	0.231	0.28	53.187	0.182	0.596	> 100	0.29
	351	10.675	0.667	33.154	0.257	60.987	0.163	0.304	54.618	0.236	0.617	> 100	0.362
	376	27.331	0.646	35.191	0.527	67.737	0.392	0.343	270.442	0.181	0.678	> 100	0.415
	394	14.667	0.944	28.24	0.852	0	1	0.312	204.626	−0.521	0.603	> 100	0.607
	413	26.189	0.543	57.219	0.365	101.593	0.233	0.355	326.708	1.242	0.847	> 100	0.299
	420	10.155	0.701	30.839	0.223	0	0.999	0.312	54.466	0.163	0.612	> 100	0.377
	434	17.897	0.487	33.021	0.627	27.894	0.72	0.195	108.6	1.129	0.625	2.532	0.406
	**451**	**20.768**	**0.47**	**35.272**	**0.155**	**61.617**	**0.083**	**0.42**	**1069.35**	**0.409**	**0.749**	**> 100**	**0.189**
	452	13.13	0.629	36.162	0.35	69.991	0.231	0.28	53.187	0.182	0.596	> 100	0.29
	457	21.089	0.456	37.313	0.205	34.689	0.254	0.405	695.092	0.446	0.739	> 100	0.299
	458	−0.076	0.742	2.219	0.962	−36.306	0.229	0.228	125.1	−0.087	0.469	0	0.67
	483	10.155	0.701	35.789	0.264	64.335	0.172	0.301	55.419	0.233	0.614	> 100	0.37
	484	10.168	0.708	36.287	0.295	67.17	0.192	0.294	54.759	0.203	0.606	> 100	0.378
	494	19.312	0.37	23.92	0.268	0	1	0.299	48.25	0.156	0.617	> 100	0.081
	517	22	0.436	35.401	0.35	37.15	0.372	0.376	396.982	0.338	0.708	> 100	0.193
NSP4 (n = 35)	58	22.397	0.57	52.737	0.414	101.92	0.277	0.357	1471.56	0.452	0.701	> 100	0.24
	79	12.691	0.681	45.098	0.412	0	0.999	0.104	62.057	0.119	0.558	> 100	0.384
	**81**	**24.742**	**0.488**	**115.241**	**0.209**	0	1	**0.363**	**1770.92**	**2.637**	**0.846**	**> 100**	**0.007**
	101	22.639	0.558	52.821	0.414	111.783	0.258	0.357	1469.49	0.436	0.698	> 100	0.141
	107	12.668	0.681	39.8	0.288	82.234	0.172	0.109	59.767	0.163	0.576	> 100	0.347
	271	−15.766	0.906	−70.469	0.55	−16.293	0.92	−0.088	54.207	−1.518	0.24	0.443	0.67
	274	12.294	0.688	54.233	0.201	0	1	0.138	67.26	0.309	0.606	> 100	0.369
	402	12.294	0.688	57.872	0.223	0	1	0.137	68.25	0.401	0.61	> 100	0.387
	477	12.2	0.706	60.703	0.371	0	1	0.121	67.898	0.337	0.586	> 100	0.345
	500	21.41	0.572	114.94	0.239	0	1	0.148	77.292	1.596	0.665	> 100	0.171
	564	−14.865	0.903	11.967	0.872	−45.947	0.412	0.06	63.455	−0.216	0.341	0.46	0.67
	591	12.294	0.688	42.821	0.274	0	0.999	0.117	61.801	0.192	0.582	> 100	0.43
C (n = 26)	81	11.61	0.804	118.063	0.694	169.467	0.644	−0.122	181.557	0.397	0.748	> 100	0.484
	89	7.514	0.704	180.354	0.337	0	1	−0.229	17.22	0.964	0.712	> 100	0.075
	92	7.842	0.666	58.084	0.587	0	1	−0.282	13.892	−0.079	0.592	> 100	0.071
E2 (n = 35)	221	13.752	0.483	62.324	0.222	0	1	0.033	26.531	1.253	0.806	> 100	0.009
	248	13.823	0.46	35.148	0.206	71.796	0.098	0.107	33.016	0.369	0.73	> 100	0.228
	255	14.02	0.447	39.135	0.149	34.002	0.212	0.143	37.092	0.556	0.769	> 100	0.091
E1 (n = 34)	213	15.819	0.446	58.038	0.223	0	1	0.121	920.418	0.62	0.759	> 100	0.288
	304	16.291	0.436	51.496	0.348	51.297	0.372	0.12	867.485	0.442	0.728	> 100	0.205
	397	23.761	0.296	74.074	0.164	111.096	0.122	0.131	25059.4	1.383	0.883	> 100	0.179

The number of sequences that included for analysis was shown. Sites were found under positive selection were labeled in bold.

**Table 3:** Positive selection sites identified by IFEL and MEME methods with p-value ≤ 0.1 or, REL methods with Bayes factor ≥50.

**CHIKV isolate**	**nsP2**													**nsP3**																**nsP4**								**C**			**E2**			**E1**	
	**67**	**768**	**13**	**14**	**72**	**77**	**224**	**238**	**328**	**332**	**351**	**376**	**394**	**413**	**420**	**434**	**451**	**452**	**457**	**458**	**483**	**484**	**494**	**517**	**58**	**79**	**81**	**101**	**107**	**271**	**274**	**402**	**477**	**500**	**564**	**591**	**81**	**89**	**92**	**221**	**248**	**255**	**213**	**304**	**397**
ChikV.India.IND-63-WB1/1963	G	N	K	N	N	S	I	S	Q	Q	G	V	M	T	N	L	L	Q	*T*	T	D	E	L	P	M	R	K	*I*	Y	K	N	D	E	Q	D	S	M	T	P	K	L	I	V	P	L
ChikV.India.I-634029/1963	.	.	.	.	.	.	.	.	.	.	.	.	.	.	.	.	.	.	.	.	.	.	.	.	.	.	.	.	.	.	.	.	.	.	.	.	.	.	.	.	.	.	.	.	.
ChikV.India.Gibbs263/1963	.	.	.	.	.	.	.	.	.	.	.	.	.	.	.	.	.	.	.	.	.	.	.	.	.	.	.	.	.	.	.	.	.	.	.	.	.	K	.	.	.	.	.	.	.
ChikV.India.IND-73-MHS/1973	.	.	.	.	.	*T*	.	N	.	.	.	.	.	.	.	.	.	.	.	.	.	.	.	.		.	.	.	.	.	.	.	.	.	E	.	.	K	.	.	.	.	.	.	.
ChikV.India.PO731460/1973	.	.	.	.	.	*T*	.	N	.	.	.	.	.	.	.	.	.	.	.	.	.	.	.	*S*	.	.	.	.	.	.	.	.	.	.	E	.	.	K	.	.	.	.	.	.	.
ChikV.Thailand.Th35/1958	.	.	.	.	.	*T*	*T*	.	.	.	.	.	.	.	.	.	.	.	.	.	.	.	.	.	.	.	.	.	.	.	.	.	.	.	.	.	.	K	.	G	.	.	.	.	.
ChikV.Thailand.AF15561/1962	.	.	.	.	.	*T*	*T*	.	.	.	.	.	.	.	.	.	.	.	.	.	.	.	.	.	.	.	.	.	.	.	.	.	.	.	.	.	.	K	.	.	.	V	.	.	.
ChikV.Thailand.1455/1975	.	.	.	.	.	*T*	*T*	.	.	.	.	.	.	.	.	.	.	.	.	.	.	.	.	.	*T*	.	.	.	.	.	.	.	D	.	.	.	.	K	.	.	.	V	.	*S*	.
ChikV.Thailand.3412/1978	.	.	.	.	.	*T*	*T*	.	.	.	.	.	.	.	.	.	.	.	.	*I*	.	.	.	.	*T*	.	.	.	.	.	.	.	.	.	.	.	.	K	.	.	.	V	.	.	.
ChikV.Thailand.6441/1988	.	.	.	.	.	*T*	*T*	.	.	.	.	.	*T*	.	.	.	*F*	.	.		.	.	.	.	*T*	.	.	*V*	.	.	.	.	.	.	.	.	*T*	K	.	.	.	V	.	.	.
ChikV.Thailand.K0146/1995	.	.	.	.	.	*T*	*T*	.	.	.	.	*I*	.	.	.	.	*F*	.	.		.	.	.	.	*T*	.	.	*V*	.	.	.	.	.	.	.	.	.	K	.	.	.	V	.	.	.
ChikV.Thailand.SV0444/1995	.	.	.	.	.	*T*	*T*	.	.	.	.	*I*	.	.	.	.	*F*	.	.		.	.	.	.	*T*	.	.	*V*	.	.	.	.	.	.	.	.	.	K	.	.	.	V	.	.	.
ChikV.Thailand.CO392/1995	.	.	.	.	.	*T*	*T*	.	.	.	.	*I*	.	.	**.**	.	*F*	.	.		.	.	.	.	*T*	G	*D*	*V*	.	.	.	.	.	.	.	.	.	K	.	.	.	V	.	.	.
ChikV.Indonesia.RSU1/1985	.	.	.	.	.	*T*	*T*	.	.	.	.	*I*	.	*I*	.	.	.	.	.		.	.	.	.	*T*	.	.	*V*	.	.	.	.	.	.	.	.	.	K	.	.	.	V	.	.	*P*
ChikV.Indonesia.IKT23574/1983	.	.	.	.	.	*T*	*T*	.	.	.	.	*I*	.	*I*	.	.	.	.	.		.	.	.	.	*T*	.	.	*V*	.	.	.	.	.	.	.	.	.	K	.	.	.	V	*I*	.	*P*
ChikV.Philippines.Hu.85.NR.001/1985	.	.	.	.	.	*T*	*T*	.	.	.	.	*I*	.	*I*	.	*M*	.	.	.	*I*	.	.	.	.	*T*	.	.	*V*	.	.	.	.	.	.	.	.	.	K	Q	.	.	V	.	.	*P*
ChikV.Philippines.PhH15483/1985	.	.	.	.	.	*T*	*T*	.	.	.	.	*I*	.	*I*	.	*M*	.	.	.	*I*	.	.	.	.	*T*	.	.	*V*	.	.	.	.	.	.	.	.	.	K	Q	.	.	V	.	.	*P*
ChikV.Malaysia.37350/2006	.	S	.	.	.	*T*	*T*	.	.	R	.	.	.	*I*	.	Q	.	R	*I*	.	.	D	.	*S*	*T*	.	.	*V*	.	R	.	.	.	.	.	.	.	K	.	.	S	L	.	*S*	*P*
ChikV.Malaysia.37348/2006	.	S	.	.	.	*T*	*T*	.	.	R	.	.	.	*I*	.	Q	.	R	*I*	.	.	D	.	*S*	*T*	.	.	*V*	.	R	.	.	.	.	.	.	.	K	.	.	S	V	.	*S*	*P*
ChikV.Malaysia.MY002IMR-06-BP/2006	.	S	.	.	.	*T*	*T*	.	.	R	.	.	.	*I*	.	Q	.	R	*I*	.	.	D	.	*S*	.	.	.	*V*	.	R	.	.	.	.	.	.	.	K	.	.	S	V	.	*S*	*P*
ChikV.Malaysia.MY019IMR-06-BP/2006	A	S	.	.	.	*T*	*T*	.	.	R	.	.	.	*I*	.	Q	.	R	*I*	.	.	D	.	*S*	.	.	.	*V*	.	R	.	.	.	R	.	.	.	K	.	.	S	V	*F*	*S*	*P*
ChikV.Malaysia.MY002IMR-06-BP/2006	A	S	.	.	.	*T*	*T*	.	.	R	.	.	.	*I*	.	Q	.	R	*I*	.	.	D	.	*S*	.	.	.	*V*	.	R	.	.	.	.	.	.	.	K	.	.	S	V	.	*S*	*P*
ChikV.NewCaledonia.NC 2011-568/2011	.	S	.	.	.	.	*T*	.	.	R	.	.	*K*	*I*	.	Q	.	R	*I*	.	.	D	.	*S*	*T*	.	.	*V*	.	R	.	.	.	.	.	.	.	K	.	.	S	V	.	*S*	*P*
ChikV.Indonesia.Leiv.Chik.1.Russia/2013	.	S	.	.	.	.	*T*	.	.	R	.	.	.	*I*	D	Q	.	R	*I*	.	.	D	.	*S*	*T*	.	.	*V*	.	R	.	.	.	.	.	N	.	K	.	.	S	V	.	*S*	*P*
ChikV.Indonesia.0706aTw/2007	.	S	N	.	S	.	.	.	.	R	.	*I*	.	*I*	.	Q	*F*	R	*I*	.	.	D	.	*S*	*T*	.	.	*V*	.	R	.	N	.	.	.	.	*T*	K	.	.	S	V	.	*S*	*P*
ChikV.Philipines.CK12-275/2012	.	S	.	.	.	*T*	.	.	R	R	.	*I*	.	*I*	.	Q	*F*	R	*I*	.	.	D	.	*S*	*T*	.	.	*V*	.	R	.	.	.	.	.	.	*T*	K	.	.	S	V	.	*S*	*P*
ChikV.Philipines.CK12684/2012	.	S	.	.	.	*T*	.	.	.	R	.	*I*	.	*I*	.	Q	*F*	R	*I*	.	N	D	.	*S*	*T*	.	.	*V*	.	R	.	.	.	.	.	.	*T*	K	.	.	S	V	.	*S*	*P*
ChikV.Philipines.CK12-686/2012	.	S	.	.	.	*T*	.	.	.	R	.	*I*	.	*I*	.	Q	*F*	R	*I*	.	N	D	.	*S*	*T*	.	.	*V*	.	R	D	.	.	.	.	.	*T*	K	.	.	S	V	.	*S*	*P*
ChikV.Philipines.CK12-921/2012	.	S	.	S	.	*T*	.	.	.	R	.	*I*	.	*I*	.	Q	*F*	R	*I*	.	N	D	.	*S*	*T*	.	.	*V*	.	R	.	.	.	.	.	.	*T*	K	.	.	S	V	.	*S*	*P*
ChikV.Philipines.CK12-340/2012	.	S	.	.	.	*T*	.	.	.	R	.	*I*	.	*I*	.	Q	*F*	R	*I*	.	N	D	.	*S*	*T*	.	.	*V*	.	R	.	.	.	.	.	.	*T*	K	.	.	S	V	.	*S*	*P*
ChikV.Philipines.JC2012.China/2012	.	S	.	.	.	*T*	.	.	.	R	.	*I*	.	*I*	.	Q	*F*	R	*I*	.	N	D	.	*S*	*T*	.	.	*V*	.	R	.	.	.	.	.	.	*T*	K	.	.	S	V	.	*S*	.
ChikV.China.China-sy/2012	.	S	.	.	.	*T*	.	.	.	R	.	*I*	.	*I*	.	Q	*F*	R	*I*	.	N	D	.	*S*	*T*	.	.	*V*	.	R	.	.	.	.	.	.	*T*	K	.	.	S	V	.	*S*	.
ChikV.Philipines.CK12-882/2012	.	S	.	.	.	*T*	.	.	.	R	D	*I*	.	*I*	.	Q	*F*	R	*I*	.	N	D	.	*S*	*T*	.	.	*V*	.	R	.	.	.	.	.	.	*T*	K	.	.	S	V	.	*S*	.
ChikV.Philipines.CK12-906/2012	.	S	.	.	.	*T*	.	.	.	R	D	*I*	.	*I*	.	Q	*F*	R	*I*	.	N	D	.	*S*	*T*	.	.	*V*	.	R	.	.	.	.	.	.	*T*	K	.	.	S	V	.	*S*	.
ChikV.Philipines.CK12-884/2012	.	S	.	.	.	*T*	.	.	.	R	D	*I*	.	*I*	.	Q	*F*	R	*I*	.	N	D	.	*S*	*T*	.	.	*V*	.	R	.	.	.	.	.	.	*T*	K	.	.	S	V	.	*S*	.
ChikV.Philipines.CK12674/2012	.	S	.	.	.	*T*	.	.	.	R	.	*I*	.	*I*	.	Q	*F*	R	*I*	.	N	D	Q	*S*	*T*	.	.	*V*	.	R	.	.	.	.	.	.	*T*	K	.	.	S	V	.	*S*	.
ChikV.Philipines.CK12-148/2012	.	S	.	.	.	*T*	.	.	.	R	.	*I*	.	*I*	.	Q	*F*	R	*I*	.	N	D	.	*S*	*T*	.	.	*V*	.	R	.	.	.	.	.	.	*T*	K	.	.	S	V	.	*S*	.
ChikV.Philipines.CK12-709/2012	.	S	.	.	.	*T*	.	.	.	R	.	*I*	.	*I*	.	Q	*F*	R	*I*	.	N	D	.	*S*	*T*	.	.	*V*	H	R	.	.	.	.	.	.	*T*	K	.	.	S	V	.	*S*	.
ChikV.Philipines.CK12-702/2012	.	S	.	.	.	*T*	.	.	.	R	.	*I*	.	*I*	.	Q	*F*	R	*I*	.	N	D	.	*S*	*T*	.	.	*V*	H	R	.	.	.	.	.	.	*T*	K	.	.	S	V	.	*S*	.
ChikV.Philipines.CK12-708/2012	.	S	.	.	.	*T*	.	.	.	R	.	*I*	.	*I*	.	Q	*F*	R	*I*	.	N	D	.	*S*	*T*	.	.	*V*	H	R	.	.	.	.	.	.	*T*	K	.	.	S	V	.	*S*	.
ChikV.Micronesia.3462/2013	.	S	.	.	.	*T*	.	.	.	R	.	*I*	.	*I*	.	Q	*F*	R	*I*	.	N	D	.	*S*	*T*	.	.	*V*	.	R	.	.	.	.	.	.	*T*	K	.	.	S	V	.	*S*	.
ChikV.Micronesia.3807/2013	.	S	.	.	.	*T*	.	.	.	R	.	*I*	.	*I*	.	Q	*F*	R	*I*	.	N	D	.	*S*	*T*	.	.	*V*	.	R	.	.	.	.	.	.	*T*	K	.	.	S	V	.	*S*	.
ChikV.Philipines.CK12-559/2012	.	S	.	.	.	*T*	.	.	.	R	.	*I*	.	*I*	.	Q	*F*	R	*I*	.	N	D	.	*S*	*T*	.	.	*V*	.	R	.	.	.	.	.	.	*T*	K	.	.	S	V	.	*S*	.
ChikV.Philipines.CK12-545/2012	.	S	.	.	.	*T*	.	.	.	R	.	*I*	.	*I*	.	Q	*F*	R	*I*	.	N	D	.	*S*	*T*	.	.	*V*	.	R	.	.	.	.	.	.	*T*	K	.	.	S	V	.	*S*	.
ChikV.Philipines.CK12-335/2012	.	S	.	.	.	*T*	.	.	.	R	.	*I*	.	*I*	.	Q	*F*	R	*I*	.	N	D	.	*S*	*T*	.	.	*V*	.	R	.	.	.	.	.	.	*T*	K	.	.	S	V	.	*S*	.
ChikV.BritishVirginlsland.99659/2014	.	S	.	.	.	*T*	.	.	.	R	.	*I*	.	*I*	.	Q	*F*	R	*I*	.	N	D	.	*S*	*T*	.	.	*V*	.	R	.	.	.	.	.	.	*T*	K	.	.	S	V	.	*S*	.
ChikV.StMartin.H20235/2013	.	S	.	.	.	*T*	.	.	.	R	.	*I*	.	*I*	.	Q	*F*	R	*I*	.	N	D	.	*S*	*T*	.	.	*V*	.	R	.	.	.	.	.	.	*T*	K	.	.	S	V	.	*S*	.
ChikV.StMartin.CNR20236/2013	.	S	.	.	.	*T*	.	.	.	R	.	*I*	.	*I*	.	Q	*F*	R	*I*	.	N	D	.	*S*	*T*	.	.	*V*	.	R	.	.	.	.	.	.	*T*	K	.	.	S	V	.	*S*	.
ChikV.StMartin.CNR20235/2013	.	S	.	.	.	*T*	.	.	.	R	.	*I*	.	*I*	.	Q	*F*	R	*I*	.	N	D	.	*S*	*T*	.	.	*V*	.	R	.	.	.	.	.	.	*T*	K	.	.	S	V	.	*S*	.

The sites with Bayes factor ≥100 were showed in italic while three site (nsP3-77, nsP3-451 and nsP4-81) positive for more than one method were showed in bold.
