# Supplementary material for: Independent Emergence of the Cosmopolitan Asian Chikungunya Virus, Philippines 2012
Source: Sci Rep. 2015 Jul 23;5:12279. doi: 10.1038/srep12279 (PMC5378875; doi:10.1038/srep12279)
Supplement: Supplementary Information [file srep12279-s1.pdf]

Independent Emergence of the Cosmopolitan Asian Chikungunya Virus, Philippines 2012

Kim-Kee Tan[1, 2], Ava Kristy D. Sy[3], Amado O. Tandoc[3], Jing-Jing Khoo[2], Syuhaida Sulaiman[2], Li-Yen Chang[1, 2], and Sazaly AbuBakar[1, 2*]

*Corresponding author: Prof. Sazaly Abu Bakar, Tropical Infectious Diseases Research and Education Centre (TIDREC), University of Malaya, 50603 Kuala Lumpur, Malaysia. Tel: +603-79675756. Fax: +603-79675757. Email: sazaly@um.edu.my

[1]Tropical Infectious Diseases Research and Education Centre (TIDREC), University of Malaya, 50603 Kuala Lumpur, Malaysia

[2]Department of Medical Microbiology, Faculty of Medicine, University of Malaya, 50603 Kuala Lumpur, Malaysia

[3]Virology Department, Research Institute for Tropical Medicine, Department of Health, FCC Compound, Alabang, Muntinlupa City, Philippines

Supplementary Table 1: List of Chikungunya virus sequences used in the analysis

Virus strains	Country	Year	Accession no.
ChikV.India.I-634029/1963	India	1963	HM045803.1
ChikV.India.Gibbs263/1963	India	1963	HM045813.1
ChikV.India.IND-63-WB1/1963	India	1963	EF027140.1
ChikV.India.PO731460/1973	India	1973	HM045788.1
ChikV.India.IND-73-MH5/1973	India	1973	EF027141.1
ChikV.Thailand.TH35/1958	Thailand	1958	HM045810.1
ChikV.Thailand.AF15561/1962	Thailand	1962	EF452493.1
ChikV.Thailand.1455/1975	Thailand	1975	HM045814.1
ChikV.Thailand.3412/1978	Thailand	1978	HM045808.1
ChikV.Thailand.6441/1988	Thailand	1988	HM045789.1
ChikV.Thailand.SV0444/1995	Thailand	1995	HM045787.1
ChikV.Thailand.K0146/1995	Thailand	1995	HM045802.1
ChikV.Thailand.CO392/1995	Thailand	1995	HM045796.1
ChikV.Indonesia.JKT23574/1983	Indonesia	1983	HM045791.1
ChikV.Indonesia.RSU1/1985	Indonesia	1985	HM045797.1
ChikV.Philippines.PhH15483/1985	Philippines	1985	HM045790.1
ChikV.Philippines.Hu.85.NR.001/1985	Philippines	1985	HM045800.1
ChikV.Malaysia.MY019IMR-06-BP/2006	Malaysa: Bagan Panchor	2006	EU703761.1
ChikV.Malaysia.MY021IMR-06-BP/2006	Malaysa: Bagan Panchor	2006	EU703762.1
ChikV.Malaysia.MY003IMR-06-BP/2006	Malaysa: Bagan Panchor	2006	EU703760.1
ChikV.Malaysia.MY002IMR-06-BP/2006	Malaysa: Bagan Panchor	2006	EU703759.1
ChikV.MY.37348/2006	Malaysia	2006	FN295483.3
ChikV.MY.37350/2006	Malaysia	2006	FN295484.2
ChikV.NewCaledonia.NC_2011-568/2011	New Caledonia	2011	HE806461.1
ChikV.Indonesia.Leiv.Chik.1.Russia/2013	Russia (Indonesia)	2013	KF872195.1
ChikV.Indonesia.0706aTw/2007	Indonesia	2007	FJ807897.1
ChikV.Philippines CK12-148/2012	Philippines	2012	In this study
ChikV.Philippines CK12-335/2012	Philippines	2012	In this study
ChikV.Philippines CK12-340/2012	Philippines	2012	In this study
ChikV.Philippines CK12-275/2012	Philippines	2012	In this study
ChikV.Philippines CK12-545/2012	Philippines	2012	In this study
ChikV.Philippines CK12 674/2012	Philippines	2012	In this study
ChikV.Philippines CK12 684/2012	Philippines	2012	In this study
ChikV.Philippines CK12-686/2012	Philippines	2012	In this study
ChikV.Philippines CK12-702/2012	Philippines	2012	In this study
ChikV.Philippines CK12-708/2012	Philippines	2012	In this study
ChikV.Philippines CK12-709/2012	Philippines	2012	In this study
ChikV.Philippines CK12-882/2012	Philippines	2012	In this study
ChikV.Philippines CK12-884/2012	Philippines	2012	In this study
ChikV.Philippines CK12-906/2012	Philippines	2012	In this study

(Continued)

ChikV.Philippines CK12-559/2012	Philippines	2012	In this study
ChikV.Philippines CK12-921/2012	Philippines	2012	In this study
ChikV.China.China-sy/2012	China (Philippines)	2012	KF318729.1
ChikV.Philippines.JC2012.China/2012	China (Philippines)	2012	KC352904.1
ChikV.Micronesia.3807/2013	Micronesia	2013	KJ451622.1
ChikV.Micronesia.3462/2013	Micronesia	2013	KJ451623.1
ChikV.StMartin.CNR20235/2013	St. Martin	2013	European Virus Archieve
ChikV.StMartin.H20235/2013	St. Martin	2013	European Virus Archieve
ChikV.StMartin.CNR20236/2013	St. Martin	2013	European Virus Archieve
ChikV.BritishVirginIsland.99659/2014	British Virgin Island	2014	KJ451624
